# Relationship of neutrophil lymphocyte ratio, monocyte lymphocyte ratio and neutrophil monocyte ratio with treatment response in pulmonary tuberculosis patients during intensive phase treatment

**DOI:** 10.1186/s12879-024-09454-2

**Published:** 2024-06-21

**Authors:** Muniza Omair, Mirza Saifullah Baig, Waqas Ahmed Farooqui, Shaheen Kousar, Muhammad Yahya Noori, Nida Zeehan, Ayesha Khan, Saman Isa, Durre Sameen Kamran, Muhammad Furqan Bari, Mehreen Mehmood

**Affiliations:** 1 Department of Pathology, Dow International Dental College, Dow University of Health Sciences, Karachi, Pakistan; 2 Department of Pulmonology, Ojha Institute of Chest Diseases, Dow University of Health Sciences, Karachi, Pakistan; 3 School of Public Health, Dow University of Health Sciences, Karachi, Pakistan; 4 Dr. Ishrat-ul-Ebaad Khan Institute of Blood Diseases, Dow University of Health Sciences, Karachi, Pakistan; 5 Department of Pathology, Dow Medical College, Dow University of Health Sciences, Karachi, Pakistan; 6 Department of Pathology, Dr. Ishrat-ul-Ebaad Khan Institute of Oral Health Science, Dow University of Health Sciences, Karachi, Pakistan; 7 Fcps Hematology Resident at Dr. Ishratul Ebaad Khan Institute of Blood Diseases, Dow University of Health Sciences, Karachi, Pakistan; 8 Department of Pathology, Dr. Ishratul Ebaad Khan Institute of Oral Health Sciences, Dow University of Health Sciences, Karachi, Pakistan; 9 Department of Pathology, Dow University of Health Sciences, Karachi, Pakistan

**Keywords:** Pulmonary tuberculosis, Neutrophil lymphocyte ratio, Monocyte lymphocyte ratio, Neutrophil monocyte ratio, Intensive phase treatment, Treatment response, Sputum Smear Microscopy

## Abstract

**Objective:**

To determine the relationship of Neutrophil Lymphocyte Ratio (NLR), Monocyte Lymphocyte Ratio (MLR), and Neutrophil Monocyte Ratio (NMR) with treatment response in Pulmonary Tuberculosis (PTB) patients during intensive phase treatment (IPT).

**Methods:**

This analytical cross-sectional study was conducted at Ojha Institute of Chest Diseases (OICD), Dow University of Health Sciences, from February to December 2021. 100 patients were enrolled using purposive sampling technique. Both male and female of age 18 and above, rifampicin sensitive newly diagnosed cases of PTB by Acid Fast Bacilli (AFB) microscopy and Gene Xpert MTB/RIF were included. SPSS version 26 was used to analyze data. Numerical data was expressed in median and interquartile range and categorical data was expressed in frequencies and percentages.

**Results:**

Out of total 100 patients, 81% (*n* = 81) showed treatment response with negative AFB Sputum Smear Microscopy (SSM) after 2nd month. Out of 81% (*n* = 81) of the patients who achieved treatment response, 83.9% (*n* = 68) also had decreased NLR, 85.2% (*n* = 69) had decreased MLR and 83.9% (*n* = 68) had decreased NMR from baseline. However 19% (*n* = 19) did not achieved treatment response with positive AFB SSM after 2nd month of ATT (Anti tuberculosis treatment), among them 10.52% (*n* = 2) were INH resistant with no decrease in all the ratios after 2nd month.

**Conclusion:**

Leukocyte ratios decreased significantly from baseline as PTB was treated in patients who achieved treatment response with negative AFB SSM after two months of ATT and hence these ratios could be used as markers to monitor the treatment response.

## Background

Tuberculosis (TB) is a significant global health threat, ranking as the second leading cause of death from an infectious agent, following COVID-19. Pakistan stands fifth worldwide for TB prevalence, emphasizing the urgent need for early diagnosis, proper treatment, and vigilant monitoring of anti-tuberculosis therapy (ATT) to mitigate TB-related morbidity and mortality. Prompt evaluation of treatment response after two months is crucial, especially in the era of rising antimicrobial resistance [1, 2].

Monitoring ATT is essential for adjusting TB treatment and preventing drug resistance [3]. The World Health Organization (WHO) recommends a combination of history, physical examination, sputum smear microscopy (SSM), and chest X-ray post intensive phase treatment (IPT) [4]. However, sputum smear has limited performance, and chest X-ray may show minimal improvement or remain unchanged [1, 5].

Leukocytes are a well-known marker reflecting inflammation, offer potential in monitoring TB progress and treatment response [6, 7]. Several researchers have discovered the possible relevance of different leukocyte ratios in following inflammatory processes e.g. cardiovascular and chronic renal disease, diabetes, hypertension, and TB [1, 8–11]. The total leukocyte count (TLC) often increases in response to various factors including inflammation, infection, trauma, and hormonal influences [12, 13].

Neutrophilia and lymphopenia, integral to these ratios, accurately reflect illness severity and predict outcomes in infectious diseases, including bacteremia [5, 14]. As TB pathogenesis involves a severe local inflammatory response with variations in white blood cell subtypes, these ratios serve as indicators of disease progress [1, 15, 16, 13].

Studies have shown the utility of leukocyte ratios, including neutrophil-to-lymphocyte ratio (NLR), monocyte-to-lymphocyte ratio (MLR), and neutrophil-to-monocyte ratio (NMR), in various bacterial infections, including TB [17]. These ratios have been used as a marker to see treatment response and severity of various diseases [11, 18, 19]. However very limited studies, which have shown that hematological parameters play an essential role in treatment strategies.

Reductions in MLR and NLR have been observed in TB patients following ATT, indicating potential as markers for disease progression [20, 21]. Additionally, higher MLR and NLR levels at pretreatment have been associated with delayed sputum smear conversion in TB patients [22]. In another studies Wang et al. [23], and Iqbal et al. [24] highlighted the role of these ratios as a marker that could predict the response of treatment in PTB. Notably, NMR has been investigated for the first time in Pakistan to monitor treatment response in newly active TB patients, suggesting its potential utility alongside other leukocyte ratios.

TB remains a major health concern globally and in Pakistan, with treatment failure posing a significant challenge. Leukocyte ratios offer a cost-effective means of monitoring TB progression and treatment response, potentially enhancing outcomes and reducing healthcare expenses.

## Methods

This was an analytical cross-sectional study performed at Ojha Institute of Chest Diseases (OICD, DUHS) in Karachi, city of Pakistan. This study was approved by DUHS, by Institutional review board (164th meeting held on 5th December 2020) with Ref. No. IRB-1983/DUHS/Approval/. The patient’s informed and thorough consents were then obtained, and two questionnaires were designed especially for this research, one for inclusion of patients and assessment at baseline and second to reassess them after two months of anti-tuberculosis treatment given them according to Standard WHO Regimen for new active cases of PTB that applies in the Ojha institute of chest diseases (OICD). We monitored baseline NLR, MLR and NMR and after determination of these ratios via CBC, WHO standard dose of ATT (Isoniazid, Rifampicin, ethambutol and pyrazinamide) was given to the patients and reassessed them after 2 months of treatment. 100 samples were collected from OICD, DUHS. Laboratory work was done in Dr. Ishrat-ul-Ebaad khan Institute of Blood Diseases, (DUHS).

### Inclusion criteria

This study was conducted from February to December 2021, at OJHA institute of chest disease, Karachi. We included total of 100 newly diagnosed PTB patients who were diagnosed by AFB sputum smear microscopy and Gene Xpert (PCR). Patients were both male and female with age between 18 and 75 years. All patients were AFB sputum smear positive and rifampicin sensitive.

### Exclusion criteria

Patients having other chronic inflammatory disease such as SLE, sarcoidosis, connective tissue disorder, having H/O cardiovascular disease, BMI 27.5kgm^2^ or above, known diabetes, hypertension, and current smokers were excluded by performing HbA1c, lipid profile and antibody testing in all the suspected cases.

### Statistical analysis

Sample size was 75 and calculated by using PASS version 15 (NCSS, Kaysville, Utah, USA), by taking an article as a reference [24]. In this article there was an independent t test was applied with 95% C.I and 80% power of the test, mean SD of monocyte lymphocyte ratio in control group was (0.24 *±* 0.7) and in TB group was (0.2 *±* 0.10) at 2nd month by one sample proportion, with 95% confidence of interval, 80% power of the test. The power of the test was calculated to justify the sample size of 100 samples using PASS version 15 software, based on a chi-square test with 95% confidence of interval, an effect size of 0.287449 with degrees of freedom (df) 1 computed using results from an association between NLR and treatment response. The power of the test was found for all parameters more than 80% using results from an association of MLR with treatment response (effect size = 0.251409, df = 1), NMR with treatment response (effect size = 0.385497, df = 1). We included 100 patients just to offset the effect of missing patients.

In the first stage we assessed already diagnosed PTB patients by taking a detailed history and physical examination i.e. weight, height and blood pressure record (at least 3 times) were taken and following sign and symptoms were noted by using Proforma which was designed for this research. Cough, fever, shortness of breath (SOB), chest pain and weight. Initially at baseline (before starting ATT), 3 ml of blood was collected from chest clinic, (OICD) in EDTA tube (purple top) and CBC was done by automated hematology analyzer (Sysmex XN-9000). All procedures and protocols are documented and followed. External QC is ISO 15,189 is in place. Regular internal and external audits to ensure quality assurance was done on daily basis.

From Absolute WBC counts, NLR, MLR and NMR was calculated by applying formulae given below in Eq: 1, 2, and 3 [25]:

1 $$NLR = \frac{{{\rm{Absolute\ number\ of\ neutrophils}}}}{{{\rm{Absolute\ number\ of\ lymphocytes}}}}$$

2 $$MLR = \frac{{{\rm{Absolute}}\,{\rm{number}}\,{\rm{of}}\,{\rm{monocyte}}}}{{{\rm{Absolute}}\,{\rm{number}}\,{\rm{of}}\,{\rm{lymphocytes}}}}$$

3 $$NMR = \frac{{{\rm{Absolute}}\,{\rm{number}}\,{\rm{of}}\,{\rm{neutrophils}}}}{{{\rm{Absolute}}\,{\rm{number}}\,{\rm{of}}\,{\rm{monocyte}}}}$$

After determination of pre intensive phase treatment NLR, MLR and NMR via CBC, WHO standard dose of ATT (Isoniazid, Rifampicin, ethambutol and pyrazinamide) for new active cases of PTB was started for 2 months. After completion of intensive phase treatment again CBC of these patients were done and their ratios was calculated in the same way as previous and patients were reassessed by using second designed research Performa via improvement in clinical symptoms, weight gain and AFB sputum smear conversion to study the relationship between NLR, MLR, NMR and treatment response.

Successful TB treatment response is assessed after 2 months of IPT in PTB on the basis of: Conversion of AFB sputum smear, any weight gain from baseline and improvement in any of clinical symptoms (fever. cough, SOB and chest pain). AFB sputum conversion after 2nd month of ATT was mainly considered as treatment response criteria in our study. However no treatment response is defined as persistent positive sputum SSM results alone OR in not expectorating patients, persistence of fever and weight loss after completion of 2 months IPT. Likewise Improvement in ratio is defined as the decreased ratios or moving towards normal after ATT in comparison to baseline values after ATT and no improvement in ratio is defined as increased ratios after ATT in comparison to baseline values or when after ATT, values remained same as baseline.

IBM SPSS version 26 was used to analyze data. Categorical data expressed in percentages, and the median and interquartile range (IQR) was used for numerical data. Chi square and binary logistic regression analysis was applied to see the relationship of these ratios with treatment response. Shapiro wilks test was used to check normality of data. Statistically *P*-value of 0.05 or less was considered as significant.

## Results

### Demographic data of research subjects

Total of 100 patients had median age of 32 years, minimum age was 18 years and maximum was 75 years with 52% (*n* = 52)of them being male and 48% (*n* = 48) were female. Before treatment overall median (IQR) BMI (Kg/m^2^) was 17.6 (15.5–20.1), weight (kg) was 45.0 (41.0–53.5). After treatment overall median (IQR) weight (kg) was 47.0 (41.0–55.5). Among 100 patients, treatment response was achieved by 81% (*n* = 81) of the patients however 19% (*n* = 19) of the patients did not achieve treatment response with negative AFB SSM after 2nd month of ATT. 86% (*n* = 86) of the patients showed improvement in symptoms, 69% (*n* = 69) of the patients gained weight median (IQR) of 1 (1–2) Kg and 81% (*n* = 81) of patients showed negative AFB SSM results after two months treatment. Median (IQR) NLR at baseline and after intensive phase treatment was observed to be 3.89 (2.86–5.98) and 2.11 (1.58–3.47), MLR 0.54 (0.35–0.73) and 0.33 (0.24–0.46), NMR 7.59 (6.02–9.50) and 6.59 (5.71–8.62) respectively. (Table 1)

**Table 1:** Frequency of improvement in leukocyte ratios as compared to baseline in PTB patients with treatment response

Parameters	Total
Treatment Response with conversion of AFB conversion	81 (81%)
No Treatment Response	19 (19%)
**Improvement after ATT**	**N = 100 (%)**
MLR	81 (81)
NMR	77 (77)
NLR	79 (79)
**Treatment response and Improvement**	**N = 81 (%)**
NLR	68 (83.9)
MLR	69 (85.2)
NMR	68 (83.9)

NLR, Neutrophil Lymphocyte Ratio; MLR, Monocyte Lymphocyte Ratio; NMR, Neutrophil Monocyte Ratio; ATT, Anti Tuberculosis Treatment

We examined relationship between NLR, MLR and NMR with treatment response by using Chi-square test. There is a significant (*p* < 0.05) relationship between these leukocyte ratios and treatment response was observed. 81 (81%) out of 100 patients showed treatment response. With treatment response 83.9% (68 out of 81) of patients showed improvement in NLR (decreased as compared to baseline value or moving towards normal), 85.2% (69 out of 81) of patients showed improvement in MLR (decreased), 83.9% (68 out of 81) of patients showed improvement in NMR (decreased). Total of 19 patients showed no treatment response among them 42.1% (8 out of 19) of patients showed no improvement in NLR. 36.8% (7 out of19) of patients showed no improvement in MLR. 52.6% (10 out of 19) of patients showed no improvement in NMR. (Table 2)

**Table 2:** Relationship between Leukocyte Ratios with Treatment Response

Parameters	Treatment Response in PTB Patients *N* = 81 (%)	No Treatment Response in PTB Patients *N* = 19 (%)	*P*-value^£^
**NLR**			
Improved	68 (84.0)	11 (57.9)	0.01*
Not improved	13 (16.0)	8 (42.1)	
**MLR**		Data	
Improved	69 (85.2)	12 (63.2)	0.02*
Not improved	12 (14.8)	7 (36.8)	
**NMR**			
Improved	68 (84.0)	9 (47.4)	0.01*
Not improved	13 (16.0)	10 (52.6)	

^£^Chi-square test for association, NLR, Neutrophil Lymphocyte Ratio; MLR, Monocyte Lymphocyte Ratio; NMR, Neutrophil Monocyte Ratio, PTB, Pulmonary Tuberculosis

Among total 100 patients, 2 patients had decreased NLR only; one of them achieved treatment response while other did not achieve treatment response. For MLR 8 patients had decreased MLR only, among them 4 had achieved treatment response while 3 did not achieve treatment response. For NMR 3 patients had decreased NMR only, two of them achieved treatment response while one patient did not achieve treatment response. (Table 3)

**Table 3:** Frequency of patients who had decreased ONE parameter Only

Parameter	Decreased only	Achieved treatment response	No treatment response
NLR	2	1	1
MLR	8	4	3
NMR	3	2	1

NLR, Neutrophil Lymphocyte Ratio; MLR, Monocyte Lymphocyte Ratio; NMR, Neutrophil Monocyte Ratio

We also compared the difference of the ratio changes by applying independent t-test. It reports the comparison for difference in NLR, MLR and NMR for patients with treatment response. Results showed patients with treatment response “yes” had mean NLR difference 2.90 (SD = ± 3.33), mean MLR difference 0.35 (SD = ± 0.31) and mean NMR difference − 1.67 (SD = ± 4.10), whereas patients with “No” treatment response had mean NLR difference 2.39 (SD = ± 1.98), mean MLR difference 0.30 (SD = ± 0.36) and mean NMR difference − 5.40 (SD = ± 2.38). Independent sample t-test showed a significant mean difference of NMR between treatment response “yes” vs. “No” patients (*p* < 0.001). (Table 4)

**Table 4:** Difference of ratio changes before and after anti tuberculosis treatment

Variables	Treatment Response				*p*-value
	Yes *(n = 81)*		No *(n = 19)*		
	Mean	SD	Mean	SD	
Difference NLR	2.90	3.33	2.39	1.98	0.52
Difference MLR	0.35	0.31	0.30	0.36	0.55
Difference NMR	-1.67	4.10	-5.40	2.38	< 0.001*

Independent t-test for difference of ratio changes, NLR, Neutrophil Lymphocyte Ratio; MLR, Monocyte Lymphocyte Ratio; NMR, Neutrophil Monocyte Ratio; **IQR**, Inter Quartile Range

### Binary logistic regression analysis of leukocyte ratios with treatment response

Univariate logistic regression analyses showed that patients with improved NLR after IPT as compared to baseline value, had 3.8 times more chances to achieve treatment response (OR = 3.80, 95% CI: 1.28–11.28, *p*-value = 0.01). Patients with improved MLR after IPT as compared to baseline, had 3.3 times more chances to achieve treatment response (OR = 3.35, 95%CI: 1.09–10.23, *p*-value = 0.02). Patients with improved NMR after IPT, had 5.8 times more chances to achieve treatment response (OR = 3.80, 95% CI: 2.0–17.1, *p*-value = 0.01) (Table 5).

**Table 5:** Univariate and Multivariate Analysis of Treatment Response for Leukocyte Ratios

Parameters	Univariable Analysis OR^a^ (95% CI)	*P*-value	Multivariable Analysis OR^b^ (95% CI)	*P*-value
**NLR**				
Not improved	1		1	
Improved	3.80 (1.28–11.28)	0.01	3.4 (1.10–11.7)	0.05
**MLR**				
Not improved	1		1	
Improved	3.35 (1.09–10.23)	0.02	4.31 (1.17–15.81)	0.03
**NMR**				
Not improved	1		1	
Improved	5.81 (2.0-17.1)	0.01	4.86 (1.49–15.78)	0.01

OR^a^: Unadjusted odds ratios; OR^b^: Odds ratios adjusted for all independent variables using binary logistic regression; C.I, Confidence Interval

## Discussion

The study aimed to compare the NLR, MLR, and NMR in PTB patients before and after intensive phase treatment, and to correlate these ratios with treatment response. The results showed a decrease in the ratio of patients who achieved sputum smear negativity following ATT, indicating treatment efficacy, especially in resource-constrained settings.

Complete Blood Count (CBC) is frequently performed in clinical practice [21]. Reversible peripheral blood abnormalities are frequently linked with PTB, and these hematological changes have been served as diagnostic, prognostic and therapeutic response markers [26]. In this study we observed the patterns of WBCs ratios and their relationship with treatment response.

Chi-square tests revealed a significant correlation between these ratios and treatment response, regardless of age or gender. Monitoring AFB sputum conversion into ATT every two months was identified as critical for assessing treatment efficacy.

Similar to the findings of Stanikzai et al., 81% of patients responded to treatment, while 19% did not respond, which corresponded to positive sputum smear results after two months of ATT [27].

Among the respondents, 83.9% showed improved ratios and AFB sputum smear conversion. Notably, 10.52% of non-responders showed INH resistance with no ratio improvement, indicating that the test could be useful in detecting mycobacterial resistance by the second month of treatment. MLR has been identified as a possible predictor of bloodstream infections and drug resistance [11]. Naranbhai et al. suggests that NLR and MLR play a predictive role in detecting early disease severity and mortality [18, 28, 29].

Elevated MLR, NLR, and NMR were associated with positive sputum after two months of ATT [22], consistent with previous findings linking high pretreatment MLR with delayed sputum conversion [21]. Although some patients didn’t show ratio improvement, they had positive outcomes at the end of the second and sixth months of treatment.

MLR and NLR are expected to be elevated during active disease and normalize with effective treatment, serving as predictive tools for TB [20, 21].

The MLR, NLR, and NMR ratios increase in chronic inflammations such as TB and decline when ATT is given. Numerous investigations have discovered a relationship between TB and high MLR and NLR, which, following 2 months of the intensive and continuation phases of treatment, drop and return to normal with ATT from the baseline value [20, 21]. This implies that MLR and NLR should be high while the disease is active and normal or low when the patient has a healthy immune system. They could thus be used as a TB forecasting tool [11, 21, 23, 24].

we assessed the relationship between improvement in these ratios and treatment response and we discovered that after therapy, all of these ratios decreased significantly, implying that when the bacterial burden decreases, the levels revert to lower values in patients who became SSM negative hence, indicated that there is a relationship between improvement in these ratios after ATT and treatment response as proven by chi square. This reliance could imply that assessing these ratios before and after therapy can help predict clinical outcome. This set of ratios could be used as predictors for sputum smear negativity after 2nd month of ATT, as proven by logistic regression in a model for predictors. These findings could imply that monitoring these predictors is a useful indicator of treatment response assessment. MLR, NLR, and NMR levels reduced significantly after ATT, as described above, and matched those seen in other research.

### Limitations of the study

Due to budget and time constraints, our study did not use MTB culture for diagnosis, despite its significance for diagnosis and monitoring. While GeneXpert detected Rifampicin sensitivity, other drug resistances may exist, necessitating MTB culture and the Drug Sensitivity Test (DST). We did not follow-up with patients after six months of treatment due to high dropout rates, which limited our understanding of leukocyte ratio dynamics and TB progression prediction. Furthermore, our small sample size, which is limited by financial and time constraints, impedes thorough analysis. Future research should validate these findings by comparing ratios across multiple treatment stages.

## Conclusion

Leukocyte ratios collected from routine CBC tests are helpful in identifying PTB progress, according to our research. They are inexpensive, simple, and convenient markers. In order to provide more reliable findings, this test should be used along with the WHO-recommended tests to evaluate the effectiveness of ATT. However, more research is still required to back up these findings.

## Data Availability

Dow University of Health Sciences has ownership rights to the major databases (DUHS). The Ojha institute of chest illnesses has given the corresponding author permission to make the datasets created and/or analyzed during the current investigation available upon reasonable request (DUHS).

## Declarations

It is declared that the study was conducted by the approval of Dow University of Health Sciences (DUHS) institutional review board (164th meeting held on 5th December 2020) with ref #: IRB-1983/DUHS/Approval/2021of institutional Review Board (IRB), and all the relevant guidelines and regulations were followed.

## Abbreviations

(NLR): Neutrophil Lymphocyte Ratio
(MLR): Monocyte Lymphocyte Ratio
(NMR): Neutrophil Monocyte Ratio
(PTB): Pulmonary Tuberculosis patients
(IPT): Intensive phase treatment
(OICD): Ojha Institute of Chest Diseases
MTB/RIF: Mycobacterium tuberculosis/Rifampicin
AFB: Acid Fast Bacilli
SSM: Sputum Smear Microscopy
INH: Isoniazid
SSM: Sputum smear microscopy
TLC: Total leucocyte count
NC: Neutrophil Counts
WHO: World Health Organization
CXR: Chest X-ray
RTI: Respiratory tract infection
UTI: Urinary tract infection
MDR- TB: Multidrug resistant Tuberculosis
BMI: Body mass index
EDTA: Ethylene diamine acetic acid
SOB: Shortness of Breath
